# In Vitro Combination of Ascorbic and Ellagic Acids in Sperm Oxidative Damage Inhibition

**DOI:** 10.3390/ijms232314751

**Published:** 2022-11-25

**Authors:** Filomena Mottola, Concetta Iovine, Maria Carannante, Marianna Santonastaso, Lucia Rocco

**Affiliations:** 1 Department of Environmental, Biological and Pharmaceutical Sciences and Technologies, University of Campania “Luigi Vanvitelli”, 81100 Caserta, Italy; 2 Clinical Analysis Laboratory Gamma, 81100 Caserta, Italy; 3 Department of Woman, Child and General and Special Surgery, University of Campania “Luigi Vanvitelli”, 80138 Napoli, Italy

**Keywords:** antioxidants, reactive oxygen species, DNA fragmentation, male infertility, genotoxicity, antigenotoxicity, sperm DNA, human spermatozoa

## Abstract

It is known that an altered redox balance interferes with normal spermatic functions. Exposure to genotoxic substances capable of producing oxidative stress (OS) can cause infertility in humans. The use of antioxidants to reduce oxidative stress contributes to the improvement in reproductive function. This study focused on an antigenotoxic evaluation of ellagic acid (EA) and ascorbic acid (AA) in combination against benzene genotoxic action on human spermatozoa in vitro. In addition to the evaluation of sperm parameters, damage in sperm genetic material and intracellular ROS quantification were assessed after AA, EA and benzene co-exposure using the TUNEL technique and DCF assay. The results showed that the combination of the two antioxidants generates a greater time-dependent antigenotoxic action, reducing both the sperm DNA fragmentation index and the oxidative stress. The genoprotective effect of AA and EA association in sperm cells lays the foundations for a more in-depth clinical study on the use of antioxidants as a therapy for male infertility.

## 1. Introduction

Infertility is defined as one year of unwanted non-conception with unprotected intercourse [1].

This is a clinical condition that affects 9% of couples in the world, in which the male factor contributes to 50% of fertility problems [2]. The causes of male infertility can be varied and stratified as congenital, acquired and idiopathic, often related to an increase in Reactive Oxygen Species (ROS) [3].

If present in a physiological concentration, ROS are essential for the proper performance of spermatic functions, such as acrosome reaction, capacitation and sperm–oocyte fusion [4]. An excessive ROS increase causes alterations in the redox signalling and control pathways, generating the well-known oxidative stress (OS). During spermatogenesis, the ability of mature spermatozoa to produce radicals is limited by the loss of cytoplasm and cytosolic organelles containing important sources of these species. Therefore, spermatozoa alone are not capable of generating a quantity of ROS that is able to cause OS, as is observed in the presence of high quantities of leukocytes and immature spermatozoa [5]. The ROS increase in seminal plasma is due to the presence of pathological conditions (e.g., infections, inflammation, congestion, varicocele, cryptorchidism, cancer) or exogenous factors (e.g., exposure to radiation, environmental pollution, improper nutrition, prolonged sexual abstinence), as well as particular lifestyles such as smoking, alcoholism and drug addiction [6,7,8,9,10,11,12]. Confirming this, high ROS levels were found in 25%–40% of seminal fluid samples from infertile men [13].

The reason that ROS excesses are dangerous for male reproductive health lies in their ability to cause structural and functional alterations in sperm cells. In fact, radical species cause membrane lipid peroxidation, protein oxidation and DNA damage [8,10,14,15,16,17], altering both the motility and the morphology of male gametes [9,18,19]. ROS-induced sperm DNA damage results in reduced fertilization capacity and the accumulation of genetic mutations that can lead to early miscarriages or, in some cases, transmission of genetic defects in the offspring [8,20].

ROS overproduction is always accompanied by a weakening of the antioxidant defenses of the seminal plasma. Antioxidant molecules, being, by definition able to prevent or cancel the ROS oxidizing action, could represent a turning point in the clinical management of the infertile male.

Evidence in the literature shows how ascorbic acid (AA) significantly counteracts the negative factors contributing to infertility. Also known as Vitamin C, AA is a water-soluble compound with a structure similar to six-carbon sugars and is mainly found in citrus fruits, strawberries, kiwis and tomatoes, and more generally in fruit and in fresh vegetables [21,22]. It is a major antioxidant in seminal plasma [23]. Higher concentrations of AA have been found in the seminal plasma of fertile men, leading to the assumption that it plays an important role as a seminal antioxidant [24,25]. Some studies noted an increase in semen parameters such as motility, concentration, morphology and DNA integrity, as well as higher pregnancy rates, after AA antioxidant therapy [26,27,28,29,30,31,32,33,34,35,36].

Others, on the contrary, have not found any improvement and/or have even noticed detrimental effects of vitamin C supplementation such as an increase in sperm DNA decondensation [36,37,38]. However, a reduction in OS levels in human spermatozoa exposed to AA has been confirmed by other in vitro studies [39,40]. Ellagic acid (EA), the dimeric form of gallic acid present mainly in red fruits and walnuts, can prevent oxidative damage of DNA both in vitro and in vivo [41]. Our previous study showed EA’s ability to protect human sperm cells from oxidative damage by limiting the generation of intracellular ROS [42]. In animal models, EA improved post-thawed sperm quality in broiler breeder roosters [43]. Furthermore, by suppressing OS, EA reduced sperm abnormalities cisplatin induced in rats, also showing a protective effect in epididymal, testis and prostate [44].

A more recent study confirms EA’s protective effect against radical forms, inhibiting oxidative damage provoked by phthalate in the reproductive system of rats [45].

Whereas it is well known that many antioxidants can enhance their protective action if they act in synergy, [26,46,47,48] the aim of this work was to test the potential antigenotoxic of AA and EA in combination on human spermatozoa in vitro. For this purpose, human sperm cells were treated in vitro with associated AA, plus EA and benzene as oxidizing agents, for different lengths of exposure (45, 60 and 90 min). Following the treatment, alterations in sperm cells and in genetic material were highlighted using different experimental approaches: evaluation of sperm parameters, in accordance with the guidelines of the World Health Organization 2021 (WHO) [49]; TUNEL technique, which analyzes the apoptotic process through sperm DNA fragmentation (SDF) index esteem, and intracellular ROS detection by DCF assay.

At present, there is still no information about the synergistic effects of ellagic acid and ascorbic acid on human spermatozoa; therefore, this work represents the first in vitro studies aiming to evaluate the protective and antigenotoxic action of these two molecules on male gametes.

## 2. Results

### 2.1. Semen Analysis

A total of 34 subjects with normal sperm concentration, semen volume, pH, motility and vitality (WHO, 2021) [49] were selected for the study (Table 1).

### 2.2. Sperm Parameters after Exposure to Different Substances

AA and EA, alone and in combination, did not induce any significant difference (*p* ≤ 0.05) in sperm parameters compared to the control sperm cells reported in Table 1. Their association prevented the reduction in sperm motility and viability provoked by benzene as at all exposure times, and no variation in total motility count and percentage of viable spermatozoa was observed. The addition of AA and EA did not lead to significant changes in pH.

### 2.3. DNA Fragmentation

**Figure 1:**
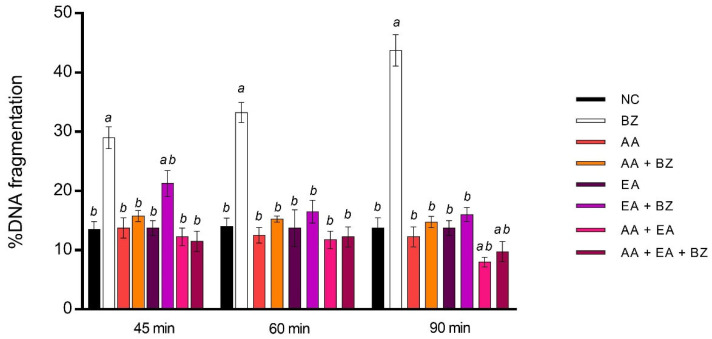
Percentage of DNA fragmentation observed in human sperm cells (n-values: 34) after 45, 60 and 90 exposure min to 0.4 μL/mL benzene (BZ), 100 μM ascorbic acid (AA), 100 μM ellagic acid (EA) and their combination. NC stands for negative control. Letters correspond to diverse statistical significances (ANOVA); a: *p* < 0.05 in comparison with NC; b: *p* < 0.05 in comparison with BZ exposure.

As already demonstrated for EA [42], AA does not induce a statistically significant percentage of DNA fragmentation index (DFI, %) after 45, 60 and 90 min of treatment, showing a damage rate below the 30% fertility threshold [50]. Unlike EA, which is capable of contrast benzene-induced damage starting from 45 co-exposure min, the association between AA and benzene shows no statistically significant damage for any treatment time. The co-exposure of EA and AA led to recorded DFI% values equal to the negative controls for 45 and 60 min. EA and AA in combination reduces DFI% with respect to negative controls after 90 min of exposure. The combination of AA and EA in association with benzene does not induce a statistical variation with respect to the negative controls (Figure 1).

### 2.4. Intracellular ROS Detection

**Figure 2:**
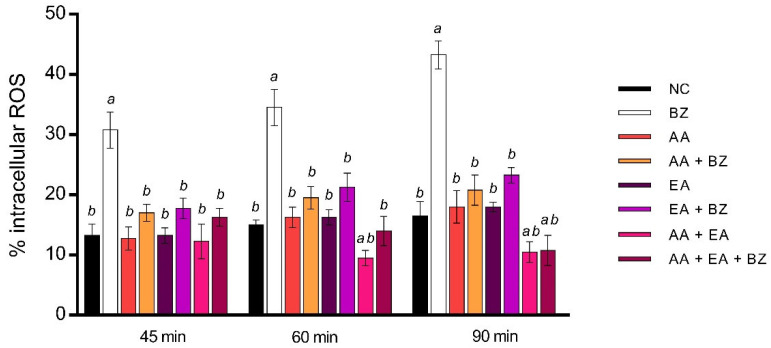
Percentage of intracellular reactive oxygen species (ROS) observed in human sperm cells (n-values: 34) after 45, 60 and 90 exposure min to 0.4 μL/mL benzene (BZ), 100 μM ascorbic acid (AA), 100 μM ellagic acid (EA), and their combination. NC stands for negative control. Letters correspond to diverse statistical significances (ANOVA); a: *p* < 0.05 in comparison with NC; b: *p* < 0.05 in comparison with BZ exposure.

DCF assay showed no statistically significant increase in intracellular ROS in sperm cells after single AA and EA exposure. AA and EA association showed no statistically significant ROS increase after 45 min, while, after 60 and 90 minutes of treatment, the ROS percentage was lower than the negative controls. The association of AA with EA and benzene reported ROS values equal to the negative controls after 45 and 60 min. Ninety minutes of treatment with AA, EA and benzene in combination reduced ROS percentage with respect to negative controls (Figure 2).

## 3. Discussion

It is known that the genotoxic damage caused by ROS is responsible for DNA fragmentation, activation of endogenous caspases and endonucleases and consequent apoptosis [9,10,14,18].

During spermatogenesis, an overproduction of free radicals when spermatozoa travel through the seminiferous tubules and epididymis is the primary causative factor leading to SDF. As ROS reach the sperm nucleus through highly susceptible membranes, they modify the bases, leading to the formation of oxidized base adducts (e.g., 8-oxo-7,8-dihydro-2-deoxyguanosine (8OhdG)). 8-oxoguanine DNA glycosylase 1 (OGG1) cleaves the oxidized base adducts, creating an unstable abasic site that tends to fragment [51]. High SDF levels can impair fertility, with reduced fertilization, higher rates of spontaneous abortion and recurrent miscarriages after ART, and, in case of conception, slow embryo development [52]. In this context, the supplementation of some antioxidants could prevent oxidative damage by improving sperm quality and reproductive outcomes. Hence, antioxidants are involved in biological system protection by inhibiting the creation of new radicals, capturing free radicals to evade the chain reaction and restoring the damage induced by free radicals [53]. The antioxidants, counteracting the DNA oxidative damage caused by ROS direct action or by genotoxic substances causing radical increase, can also act as antigenotoxic agents [46,47].

The main additional antioxidants studied in clinical trials of male subfertility are vitamin E, vitamin C, carotenoids, carnitines, coenzyme Q10 (ubiquinol), cysteine and the micronutrients folate, selenium and zinc. However, the overall quality of evidence at present is low and limited to a few small randomized controlled trials [54].

In this study, human spermatozoa were treated in vitro with an oxidizing agent, benzene, in association with two antioxidant molecules, AA and EA, in order to evaluate the antigenotoxic and possibly synergistic power of the two substances. AA and EA, alone and in combination, were safe for sperm cells; in fact, no change in sperm parameters and no oxidative damage was observed in these experimental groups. It is interesting to note that AA was able to counteract the highly genotoxic action of benzene at all co-exposure times, while EA did so starting from 45 min of co-treatment with benzene. In fact the percentage of fragmented DNA and the intracellular ROS levels in AA and benzene co-exposed group were below the 30% threshold levels of sperm DNA fragmentation considered critical for fertility [50]. A slightly higher protective action operated by AA compared to EA was observed, as the AA antigenotoxic action had already started from the minimum exposure times.

It is known that many co-administered antioxidants perform a synergistic protective action [46,55,56]. It has been proven that vitamin E generates the maximum antioxidant effect in association with AA in humans [57]. We, therefore, wanted to test whether the same increased effect in the presence of AA was obtained for EA in human sperm cells. Our results showed no statistical difference in terms of DNA fragmentation and ROS percentage for all exposure times to a combination of two antioxidants plus the oxidizing agent.

While EA alone was unable to prevent benzene-induced damage for a short time, in association with AA, it appears to have an enhanced effect. Although the effect could be due to AA alone, the data showing a DNA damage, which is higher in single exposures than in co-exposures, could suggest a synergistic action of AA and EA in combination. In fact, the percentages of DFI and ROS were statistically lower than controls for prolonged times of exposure to the association between AA and EA and to that of AA, EA and benzene.

Additionally, the combined action of the two antioxidants results in a higher quality of the semen parameters, a higher survival and a higher sperm motility with respect to the single benzene exposure. 

Based on the literature knowledge, this is the first study reporting the antigenotoxic effects of combined AA and EA exposure on human spermatozoa in vitro. The novel insight that emerged from our study is the enhanced antioxidant and antigenotoxic action of ellagic acid in combination with vitamin C. Therefore, the greater ability of the two antioxidants in combination to defend DNA from ROS-induced mutations in terms of increased cell viability, reduction in the DNA fragmentation index and ROS production in spermatozoa could represent a valid strategy to protect sperm DNA from oxidative stress. EA and AA are molecules of natural origin, highlighting the importance of a correct and balanced diet. More and more scientific evidence is emerging on the key role of antioxidants in the diet diet to prevent the onset of cardiovascular, cancer and neurodegenerative diseases, as well as metabolic disorders associated with oxidative stress [58,59,60,61]. Therefore, the antioxidant diet or therapy supplementation of the two-molecule mixture could improve the subfertility associated with ROS increase and could reduce the percentage of idiopathic hypofertility over time. However, although our results can be interpreted as encouraging for male reproductive health, we must emphasize that our data are limited to an in vitro system, and that the molecular mechanisms underlying the protective effects have not been investigated. At present, the role of vitamin C in maintaining the chromatin integrity of germ cells has not yet been elucidated; however, it has been shown that Vitamin C supplementation has a beneficial effect on the meiotic arrest of mice spermatocytes exposed to BDE-209, a flame retardant used in many industrial products. This contaminant is able to cause DNA methylation leading to cell cycle arrest and increase the ROS production. More precisely, BDE-209 oxidizes ferrous ion to ferric ion, inhibiting the activity of histone demethylase, causing the accumulation of histone demethylases removes the H3K4 tri-methylation (H3K4me3), which plays a crucial role in meiotic progression. In this context, vitamin C restores the capacity of KDM5, a family member of H3K4me3, and improves DNA repair by allowing meiotic progression [62]. Moreover, the positive effects of vitamin C on sperm chromatin were observed in male partners of couples with recurrent pregnancy loss. Daily oral administration of Vitamin C could improve spermatogenesis by increasing protamine PMN1 and PMN2 expression and chromatin condensation. As low protamine gene expression may lead to sperm dysfunction, resulting in poor implantation or negative pregnancy, Vitamin C supplementation in the male partners of couples with recurrent pregnancy loss (RPL) could improve pregnancy outcomes [63].

EA upregulated the expression of genes such as StAR, 3β-HSD and 17β-HSD involved in testosterone synthesis in the testis of arthritic rats. The increase in testosterone is likely driven by the suppression of pro-inflammatory and pro-oxidant signals; in fact, MPO, TNF-α and COX-2 levels were reduced, and endogenous antioxidant levels were high. Finally, EA acts by suppressing the expression of pro-apoptotic caspase-3 [64]. It has also been reported that EA is highly effective in reducing oxidative DNA damage partly via DNA repair enzymes’ up-regulation, supporting the role of the antioxidant in sustaining genomic stability [65].

Therefore, further studies on animal models will aim to evaluate, first of all, the effects of benzene on the meiotic cycle and the recovery capacity operated by AA and its association with EA, as well as the impact of the two antioxidants on the expression of proapoptic, protamine and DNA repair proteins in germ cells of human and animal models.

Regarding the beneficial effect of antioxidant therapies on human male fertility, clinical data are still controversial, probably because the studies are too heterogeneous, some are not randomized, placebo-controlled or double-blind, and relevant parameters, such as pregnancy, are not examined [66]. However, our results, in agreement with other in vitro studies in both humans and animal models, recognize AA and EA as perfect candidates for more in-depth clinical studies aiming to clarify the exact role of antioxidants in the management of male infertility.

## 4. Materials and Methods

### 4.1. Chemicals

AA was provided by Sigma-Aldrich (St. Louis, MO, USA) (CAS number 134-03-2); EA powder was supplied by Alfa Aesar (CAS number A15722, Hydrate 97%). AA and EA stock solution (100 μM) was prepared using ethanol solvent. Benzene (CAS number 71-43-2, 99.8 purity) was provided by Sigma-Aldrich.

### 4.2. Sample Collection

A total of 34 seminal fluids selected from men between the ages of 33 and 43 referred at Reproduction Biology Laboratory (University of Campania “Luigi Vanvitelli”) were used for the study after routine semen analysis. Patients were informed about the purpose of the study and signed a written informed consent. The selection of study participants took into account dietary and other therapeutic factors. Patients under any treatment or antioxidant supplementation were excluded from this study.

### 4.3. Semen Analysis and Exposure Procedure

Semen analysis was conducted according to WHO, 2021 [49]. Sperm parameters were determined within 1 h of collection and after liquefaction. Total motility, including progressive (P) and non-progressive (NP) spermatozoa, was assessed in a counting chamber using a light microscopy (ZEISS NT6V/10W STAB), counting approximately 1000 cells for each replicate (WHO, 2021) [49]. Test-simplets^®^ stained slides (Origio; Cooper Surgical, Inc., Måløv, Denmark) were used for morphological evaluation. To evaluate cell viability, 20 µL of each group was mixed with 20 µL of eosin–nigrosin solution (SigmaAldrich, St. Louis, MI, USA). At least 250 spermatozoa were analysed and by light microscopy at 400× magnification discerning viable sperm cells (colourless) from dead sperm (red) (Table 2).

To obtain a viable and motile spermatozoon, ejaculates were purified by 45–90% double-density gradient (SPERM GRAD™; Vitrolife, Västra Frölunda, Sweden) centrifugation. After purification, each sample was divided into the following aliquots (1 × 10^6^ sperm/mL): Minimum Essential Medium Eagle (MEM, SigmaAldrich, St. Louis, MI, USA) and benzene (0.4 μL/mL); AA (100 μM); AA (100 μM) with benzene (0.4 μL/mL); AA (100 μM) with EA (100 μM); AA (100 μM) with EA (100 μM) and benzene (0.4 μL/mL). Untreated aliquot was used as negative control. Antioxidant concentrations were chosen based on our previous study and recent literature data suggesting that these antioxidants reduce OS induced in spermatozoa by improving their function [42,67]. Sperm aliquot containing only MEM was used as negative control. After exposure for 45, 60 and 90 min at 37 °C, the pellet obtained following 5 min at 1500 revolutions per minute (rpm) centrifugation was suspended in 500 μL bicarbonate buffer (PBS 1×); the sperm parameters were then re-evaluated, and the DNA fragmentation index and intracellular ROS were quantified.

### 4.4. Sperm DNA Fragmentation (SDF)

The terminal deoxynucleotidyl transferase dUTP nick-end labelling (TUNEL) test was performed using the In-Situ Cell Death Detection Kit (Roche Diagnostics) according to Santonastaso et al., 2019 [68]. The sperm cells, after fixing in 4% paraformaldehyde for 1 h, were subjected to a permeabilizing solution containing 0.1 g of sodium citrate and 0.1% of Triton X-100. 50 μL of TUNEL reaction containing 5 μL of terminal deoxy nucleotidyl transferase enzymatic solution and 45 μL of label solution were placed on each slide and left to act for 1 h in a humid chamber at 37 °C. A solution of 4′,6-Diamidine-2′-phenylindole dihydrochloride (DAPI) was used to contrast each nucleus and 1,4 diazobicyclo (2,2,2) octane solution (20×) as anti-decay fluorescence. The percentage of spermatozoa with fragmented DNA (green fluorescence) was estimated assessing 250 sperm cells per slide at 1000× magnifications using a fluorescence microscope (Nikon Eclipse E-600) equipped with BP 330–380 nm and LP 420 nm filters. Experimental groups with DFI values higher than 30% were considered strongly compromised [50].

### 4.5. DCF Assay

Intracellular ROS was evaluated by adding 13 μM of 2,7-dichlorodihydrofluorescein- diacetate to sperm suspension for 30 min at 37 °C in the dark. After washing in PBS 1× by centrifugation for 5 min at 1500 rpm, nuclei were counterstained with DAPI and observed by fluorescence microscope analyzing 250 nuclei per silde (Nikon Eclipse E-600), as in the previous section. 

### 4.6. Statistical Analysis

All experimental data were expressed as mean ± standard deviation (SD). Data among the experimental groups were compared using ANOVA test by GraphPad Prism 6 (San Diego, CA, USA). The results were considered statistically significant for *p* ≤ 0.05.

## Figures and Tables

**Table 1:** Parameters of semen fluid selected for the study expressed as mean ± SD.

Sperm Parameters	Mean ± SD
Semen volume (mL)	3.25 ± 1.31
pH	7.70 ± 0.50
Sperm concentration (×10^6^ sperm/mL)	76.62 ± 25.36
Vitality (%)	74.50 ± 6.20
Motility (%)	
Progressive (P)	32.35 ± 16.04
Non-progressive (NP)	16.25 ± 7.97

**Table 2:** Sperm motility (progressive, P, and non-progressive, NP) and sperm vitality after antioxidant and oxidant treatment. The values were expressed as mean ± SD and compared with the control group shown in Table 1. * *p* ≤ 0.05. AA: ascorbic acid; EA: ellagic acid; BZ: benzene.

Substances Concentration	Exposure Minutes	Motility (P + NP) (%)	Vitality (%)
AA 100 µM	45	48.55 ± 7.40	78.20 ± 0.30
	60	45.30 ± 6.90	72.50 ± 0.60
	90	38.50 ± 4.60	68.80 ± 2.00
EA 100 µM	45	47.00 ± 5.10	76.00 ± 1.25
	60	43.80 ± 8.50	74.30 ± 1.70
	90	38.00 ± 9.80	69.00 ± 3.50
BZ 0.4 µL/mL	45	30.00 ± 5.70 *	70.00 ± 1.50
	60	25.35 ± 6.80 *	66.50 ± 1.80
	90	17.85 ± 4.40 *	53.20 ± 2.50 *
AA 100 µM + BZ 0.4 µL/mL	45	47.00 ± 3.70	77.50 ± 3.20
	60	42.60 ± 2.40	71.35 ± 3.50
	90	38.00 ± 5.20	66.80 ± 4.20
EA 100 µM + BZ 0.4 µL/mL	45	45.80± 4.50	75.00 ± 1.00
	60	43.00 ± 6.70	72.80 ± 0.20
	90	37.60 ± 8.30	67.50 ± 2.40
AA 100 µM + EA 100 µM	45	48.00 ± 1.50	75.10 ± 0.50
	60	44.50 ± 2.65	71.00 ± 2.00
	90	38.80 ± 4.80	69.50 ± 1.50
AA 100 µM + EA 100 µM + BZ 0.4 µL/mL	45	46.90 ± 5.20	75.30 ± 2.60
	60	44.75 ± 1.50	72.60 ± 0.55
	90	40.35 ± 4.60	68.77 ± 3.45

## Data Availability

Not applicable.
